# A Case of a Spinal Extradural Arachnoid Cyst

**DOI:** 10.1155/2019/3516598

**Published:** 2019-10-17

**Authors:** Yousuke Kojimahara, Shintaro Tsuge, Keiji Hasegawa, Katsunori Fukutake, Kazumasa Nakamura, Akihito Wada, Hiroshi Takahashi

**Affiliations:** Department of Orthopaedic Surgery, Toho University School of Medicine, Tokyo, Japan

## Abstract

The patient was a 49-year-old woman with a chief complaint of hip and buttock pain that had persisted for 3 years. She visited our hospital for aggravation of the pain. Percussion tenderness of the spinous process was observed and a T1-low, T2-high cystic lesion was detected on the dorsal side of the dural canal at the 12th thoracic vertebral level on MRI performed by a previous physician. Plane CT showed severe scalloping at the same level. During laminectomy for the 11th and 12th thoracic vertebrae, a cystic lesion of about 60 × 25 mm was noted on the dorsal side of the dural canal, with a communication pathway with the cyst present near the left 12th nerve root bifurcation. This pathway was ligated and the cyst was excised. The histopathological diagnosis was an arachnoid cyst. Pain improved after surgery, and as of 10 months after surgery, the cystic lesion has not recurred. A spinal extradural arachnoid cyst (SEAC) is a relatively rare disease. This case shows that surgical ligation of a communicating tract and cystectomy are necessary and contrast-enhanced CT was useful for the identification of the position of the communication pathway before surgery.

## 1. Introduction

A spinal extradural arachnoid cyst (SEAC) is a relatively rare disease in which the arachnoid membrane prolapses through a dural defect and expands to form an extradural cyst that induces spinal cord symptoms [1, 2]. In the Nabors classification [3], spinal arachnoid cysts are classified into three types based on the location and presence or absence of nerve tissue in the cyst. A SEAC has an extradural location and contains no nerve tissue, which makes it a type 1 cyst. Here, we report a case of a patient with a SEAC that manifested with severe low back pain.

## 2. Case

The patient was a 49-year-old woman with a chief complaint of hip and buttock pain. This pain had developed without an inducer in April 2014. A cystic lesion in the spinal canal had been pointed out by a physician. The patient received conservative treatment, but pain gradually aggravated and walking and taking a supine position at home became difficult. The patient first visited our department in October 2017. She had not particularly relevant familial or past medical history.

Physical findings on admission showed percussion tenderness in the thoracolumbar junction. There is no obvious range of motion restriction in the hip joint, and the Patrick test was negative. The score on a manual muscle test of the bilateral lower limbs was 5/5, and there was no sensory disturbance or bladder and rectal disturbance. The femoral nerve stretching test (FNST) and straight leg raising (SLR) test were both negative. The patellar tendon reflex (PTR) was normal on the bilateral sides, but the Achilles tendon reflex (ATR) was enhanced on the bilateral sides.

**Figure 1:**
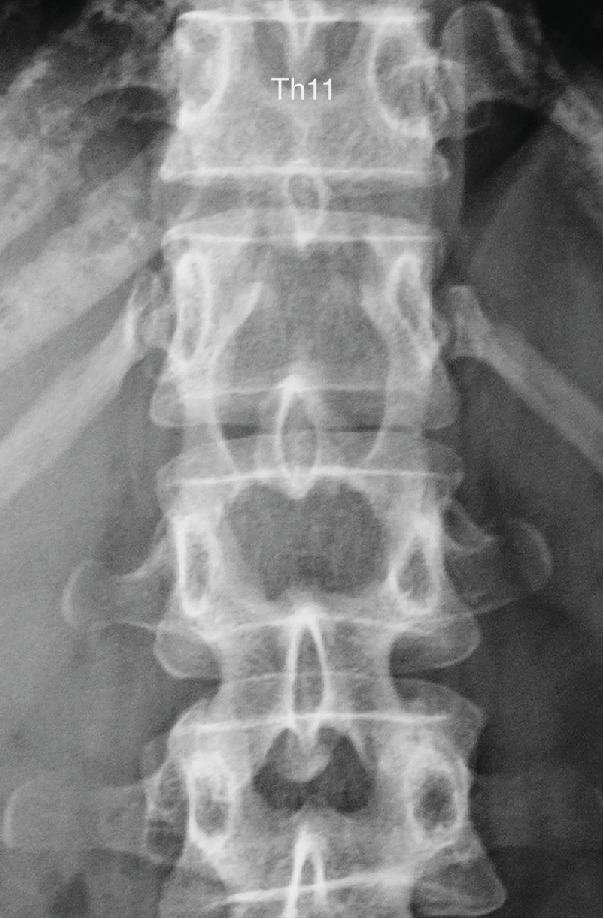
Plain radiography showed expansion of the space between the arches at T12/L1 and L1/2.

**Figure 2:**
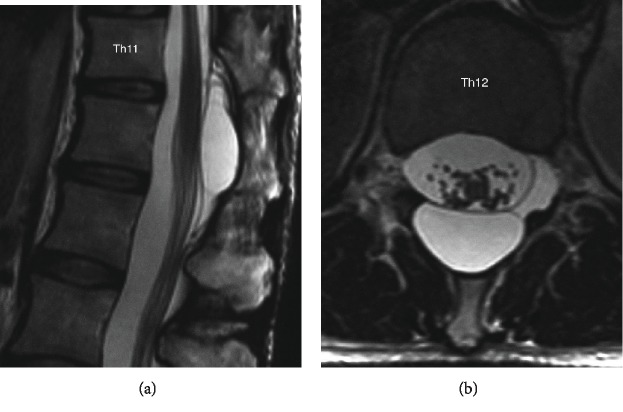
T2-weighted MRI showed a cystic lesion with T1-low/T2-high intensity at the T11-L1 level.

**Figure 3:**
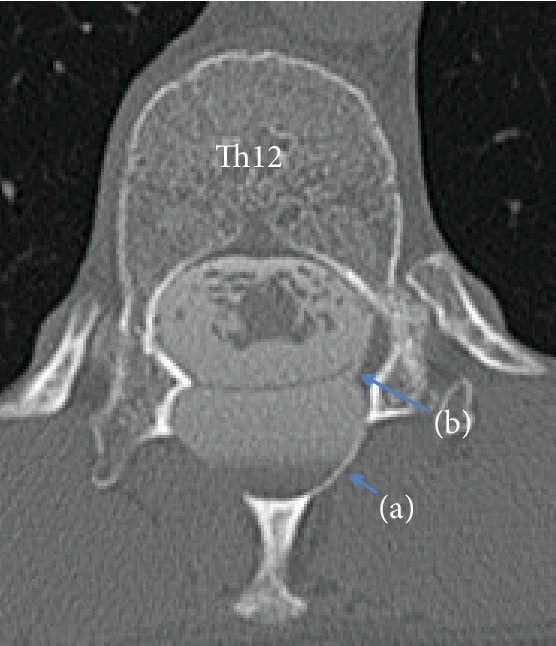
CT after myelography showed scalloping of the vertebral arch at the T12 level, with contrast medium flowing into the arch. (a) A communication pathway was present between the dural canal and the cyst at the T12 level (b).

Plain radiography showed that the T12 vertebral arch was partially lost and the space between the vertebral arches was expanded (Figure 1). MRI showed a cystic lesion with T1-low/T2-high intensity at the T11 to L1 level (Figure 2). On CT after myelography, scalloping was noted in the vertebral arch at the T12 level and contrast medium flowed into the cyst. In addition, a communication pathway between the dural canal and cyst was present at the T12 level (Figure 3).

**Figure 4:**
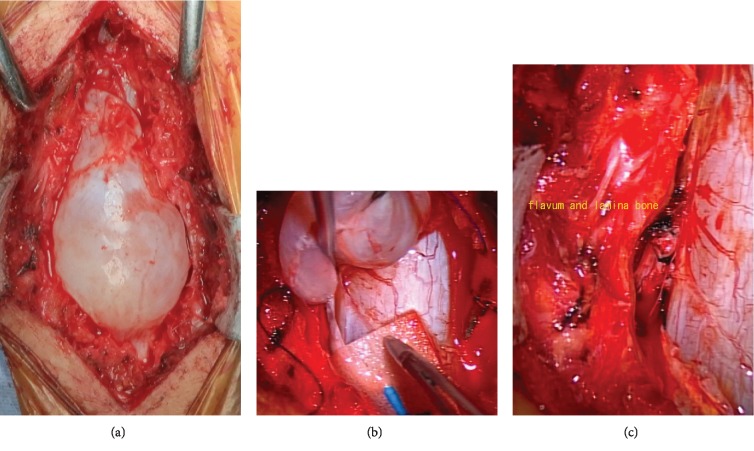
Intraoperative findings. (a) Partial loss of the bone cortex at the T12 vertebral arch. The communication pathway for cerebrospinal fluid in the axillary region of the left Th12 nerve root was ligated, and the cyst was excised.

Surgery was performed to ligate the communication pathway and excise the cyst. The anesthesia was total intravenous anesthesia (TIVA) and the body position was prone. And we use a nerve stimulator. In laminectomy for T11 and T12, the bone cortex of the T12 vertebral arch was excluded by the cyst and thinned and a bulging cyst was present just below the resected arches. When the cyst was dissected from the dura under a microscope, a communication pathway for cerebrospinal fluid was present in the axillary region of the left T12 nerve root. Thus, this region was ligated and the cyst was excised. The cyst did not adhere to the dural canal (Figure 4). Histological findings showed a cystic lesion comprised of fibrous connective tissue. Psammoma body formation in the wall and aggregation of some meningothelial cells were also observed. Low back pain rapidly resolved immediately after surgery, and there has been no recurrence of pain or of the cystic lesion on imaging for 10 months after surgery.

## 3. Discussion

SEAC accounts for 1% of spinal tumors [4], with its development in the thoracic vertebra, thoracolumbar vertebra, lumbosacral vertebra, and other sites accounting for 65%, 12%, 13%, and 10% of cases, respectively, and the cyst extending over 5 vertebrae on average [5]. In our patient, the cyst arose in the thoracolumbar vertebra and expanded over 3 vertebrae. SEAC causes neurological disorders by excluding the spinal cord and nerve root with the expansion of the cyst, but the cause of expansion is unclear; however, the check valve mechanism and secretion system have been suggested as a cause [5]. In our patient, contrast medium flowed into the cyst and no secretory cells were observed histologically, suggesting that the cause was cyst formation through the check valve mechanism.

Cho et al. showed that visualization of the communication pathway to the cyst using myelography was important for preoperative planning [6]. Similarly, in our patient, the communication pathway for inflow of contrast medium into the cyst was confirmed on myelography. This helped in making a definite diagnosis and in preoperative planning with regard to ligation of the pathway. Lee et al. reported that the recurrence rate after ligation of the communication pathway was 2%, whereas that without ligation was 66.7%, showing the importance of ligation of the pathway [5]. In our patient, total excision of the cyst and repair of the dural defect were performed, and the course has been favorable, with no recurrence of pain or the cystic lesion in 10 months postoperatively.

Excision of the cyst with obliteration of the communicating dural defect is the mainstay of treatment in symptomatic patients [7]. Concomitant fixation with excision has not previously been described, but Takagaki et al. reported that kyphosis developed in 67% of patients treated with laminectomy of multiple vertebrae and recommended the application of laminectomy to as few vertebrae as possible [8]. In our patient, we did not perform fixation, because of no facet joint damage and only two-level vertebral arch resection. But no progression of kyphosis was noted at the final follow-up.

## 4. Summary

We encountered a patient with SEAC with a chief compliant of low back pain. CT myelography was useful for diagnosis and preoperative planning. A favorable outcome was achieved by surgery for total excision of the cyst and repair of the dural defect.
